# Quality of Scar after Total Thyroidectomy: A Single Blinded Randomized Trial Comparing Octyl-Cyanoacrylate and Subcuticular Absorbable Suture

**DOI:** 10.1155/2013/270953

**Published:** 2013-11-12

**Authors:** Fabrizio Consorti, Rosaria Mancuso, Annalisa Piccolo, Eugenio Pretore, Alfredo Antonaci

**Affiliations:** Department Surgical Sciences, University “Sapienza” of Rome, Viale del Policlinico, 00161 Rome, Italy

## Abstract

*Objective*. To compare the quality of resulting scar at 6 weeks after total thyroidectomy with the use of the tissue adhesive octyl-cyanoacrylate or subcuticular absorbable suture for the closure of cervicotomy. *Material and Methods*. There are 50 patients undergoing a cervicotomy for total thyroidectomy. Twenty-five patients were randomly assigned to closure with tissue adhesive and 25 with subcuticular absorbable suture. At week 6 the scar was evaluated by blinded assessors with the Italian version of POSAS questionnaire, a validated wound scale composed of an observer's and a patient's subscale. *Results*. Assessment of scar appearance showed a statistically significant difference (*p* = 0. 038) in favor of subcuticular suture with respect to tissue adhesive on observer's assessment. The difference on patients' self-assessment was not significant. A multivariate analysis of six qualitative features of scars showed a significant influence on assessment for hyperpigmentation and relief of scar. The Italian version of POSAS proved to be reliable. *Conclusion*. Though tissue adhesive represents a valid method of skin closure, subcuticular absorbable suture provides a better aesthetic outcome in small cervical incisions in the early phase after thyroid surgery.

## 1. Background

Aesthetic outcome is particularly relevant in thyroid surgery since patients are mostly women and young adults and since the incision is in a highly sensitive and visible anatomic location. Cosmetic concern about the final scar appearance contributed to motivate the development of minimally invasive approaches for thyroid surgery and parathyroid surgery over the last decade [1–3].

Minimally invasive thyroid surgery techniques (MIT) are different but all share the same goals: reduction of tissue trauma, early hospital discharge, and better neck wound cosmetic appearance, while maintaining the same surgical outcome as traditional thyroidectomy [4].

In addition to MIT, methods of skin closure contribute to the overall aesthetic outcome and patient's satisfaction. Methods of skin closure vary in published series and are largely the results of surgeon's choice based upon the need for a rapid, economic, and reproducible technique [5].

Skin closure techniques include the use of the tissue adhesive octyl-cyanoacrylate, introduced 15 years ago in clinical practice as an ideal system of wound closure [6].

Many studies showed that tissue adhesive is an acceptable alternative to standard wound closure since it yields similar clinical and aesthetic results, even if early wound dehiscence occurs in the 1% to 5% of cases [7, 8]. In their studies, Bruns et al. [9] and Bozkurt and Saydam [10] reported cosmetic outcome in the cyanoacrylate group to be as good as or better than cosmetic outcome in the suture groups. Quinn et al. [11], Ridgway et al. [5], and Pronio et al. [12] observed no difference between closure with metal clips and closure with tissue glue. 

Furthermore, a recent study comparing adhesive strips and subcuticular absorbable suture in neck incision found an equal overall wound appearance after 6 weeks [13]. On the contrary, an Italian study [14] showed a worse aesthetic result after thyroidectomy for the tissue adhesive group.

Although octyl-cyanoacrylate has been evaluated against several suture methods in randomized clinical trials, no comparison between tissue glue and subcuticular absorbable suture as a control method has been performed following cervicotomy.

The aim of this study was to compare the aesthetic appearance of cervical incision closed with octyl-cyanoacrylate versus subcuticular absorbable suture. As a secondary aim, we validated an Italian version of the Patient and Observer Scar Assessment Scale (POSAS) [15], a well-known evaluation tool for scars. The use of a validated instrument is important to warrant reproducible results, but many of the quoted studies were based on nonvalidated subjective assessment scales.

## 2. Material and Methods 

### 2.1. Trial Design

The study was considered a single blind randomized trial because the type of wound closure used was evident from its external appearance in the early phase of the postoperative period, and we could not assume patients did not see their wound during that period, influencing their judgment of the wound. The observers were blinded to treatment.

### 2.2. Patients and Interventions

A series of consecutive patients undergoing cervicotomy for a total thyroidectomy from January 2012 to March 2013 was considered. The only inclusion criterion was being a candidate to a total thyroidectomy, regardless of the diagnosis. The exclusion criteria were previous surgery of the neck, known allergies to chemical products, history of hypertrophic scars or keloids, and poor linguistic Italian skill, preventing patients to clearly understand and answer the question of the POSAS. 

The operation was performed through a minimal open cervical incision, as described in [16]. Surgery was performed by the same surgeon (AA). The incision length was between 3.5 and 6 cm (mean = 4.13 ± 0.79). Meticulous hemostasis was achieved before approximation and closure of skin layers. Skin closure was applied after reapproximating the strap muscles of the neck and closing deep subcutaneous layer with an interrupted absorbable suture. After the operation, patients received a standard postoperative protocol and analgesic regime. 

Enrolled patients were randomly assigned through a series of random numbers generated by an electronic worksheet to have their wound closed with octyl-cyanoacrylate or subcuticular absorbable suture performed with an absorbable 3.0 suture of polyglactin. The surgeon was informed of the allocation of each patient to a type of method only at the moment of closure.

### 2.3. Outcome and Measure

The primary endpoint was the appearance of wound at the 6th post-operative week assessed through a validated scar assessment scale, the Patient and Observer Scar Assessment Scale (POSAS) [15].

POSAS was initially developed for burn scars, but it has been used and validated for several different types of wounds [17–21]. It is composed of two subscales, the Observer Scale (OSAS) and the Patient Scale (PSAS). OSAS is a 6-item and 10-grade Likert scale, graded from 1 (normal skin) to 10 (worst scar imaginable). PSAS is a 6-question scale exploring patients' opinion about their scar on a 10-grade Likert scale. The high the score the worse the appearance of the scar, with a best possible score of 6 and a worst possible score of 60 for both subscales. Both OSAS and PSAS have a 7th item about the overall opinion graded on a 10-grade Likert scale as well.

OSAS was assessed by two observers, who were trained in the use of the instrument on a series of patients before the start of this study, until they achieved a good level of agreement. To avoid an excessive burden of patients, each patient was assessed by only one of the two observers. Each observer assessed the same number of patients. PSAS was assessed during a follow-up visit with the patient in front of a mirror, while one of the observers was asking the questions.

### 2.4. Sample Size and Statistical Methods

Sample size was determined considering an expected mean POSAS score of 11 (according to [21, 22]), a smallest detectable difference of 2 mean points, a power of 80%, and a confidence interval of 95% (corresponding to a *p* threshold <0.05). With these parameters, the computed sample size consisted of two groups of 50 patients each. 

Data were analyzed by SPSS software comparing the mean value of the score of each group with the Mann-Whitney *U* test, because data were ordinal and we did not assume that the scores were normally distributed. Correlation between variables was expressed as Pearson correlation coefficient; a backward multivariate regression model was used to explore the contribution of each item to the score of the 7th item on overall assessment both for OSAS and for PSAS. 

Reliability of the Italian version of POSAS was expressed as internal consistency with the Cronbach alpha coefficient.

### 2.5. Ethical Issues

Informed consent was obtained from patients during a preoperative assessment visit. The study had ethical approval by the department. An approval from the Ethical Committee was not asked, because the two techniques of closure were already in routine use.

Since there was no hypothesis of prevalence of one treatment over the other one, a check point was established after the assessment of half of the expected patients to run an interim statistical analysis and detect possible reasons to stop the trial. 

## 3. Results

**Figure 1:**
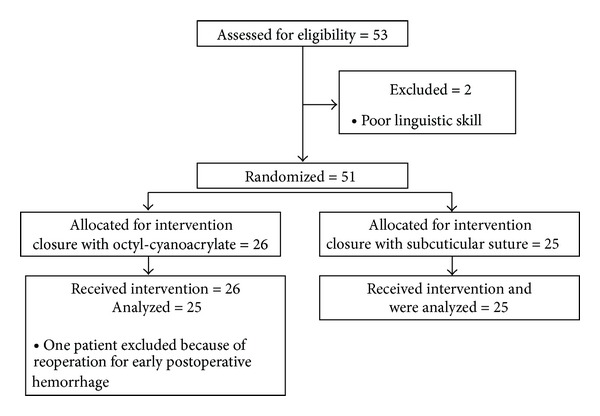
Flow of selection and randomization of patients.

At the time of check point, 53 patients were considered eligible, but three of them were excluded. In the assessed 50 patients, a highly significant difference was observed between the two groups in OSAS score, so the study was interrupted for ethical reasons after enrolling 25 + 25 patients (Figure 1). The computed power of the study with this sample size and the observed difference was 0.85. 

Table 1 shows personal and clinical data of this set of patients. No differences were observed between the two groups as to personal and clinical data of patients.

### 3.1. OSAS

Subcuticular suture was assessed more favorably with a mean score of 10.17 (±3.8) versus 13.29 (±4.4) for octyl-cyanoacrylate. This difference was statistically significant (*p* = 0.038).  Table 2 shows the distribution of scores for the different items. The best predictive multivariate regression model accounted for 71% of variance (corrected *R* ^2^ = 0.71). Although in monovariate analysis vascularity, pigmentation, and relief scores were significantly different, in the regression model only the latter two contributed in a significant way to the overall opinion of observers (Table 3). 

The Italian version of the OSAS displayed a good reliability with a Cronbach alpha value of 0.88. As a further element of reliability a strong correlation was observed between the total score (i.e., the sum of the scores of the 6 items) and the score of the 7th item on the overall opinion (Pearson *r* = 0.84).

### 3.2. PSAS

A significant difference was not observed as to PSAS. The mean value for subcuticular suture was 11.44 (±11.5) versus 11.00 (±9.8) for octyl-cyanoacrylate.  Table 4 shows the distribution of scores for the different items. The best predictive multivariate regression model in this case was slightly less powerful than for OSAS, as it accounted for 61% of the variance. The significantly contributing factors were pain, color, and thickness (Table 3). No difference was observed between the total score of men (11.55 ± 9.9) and women (11.48 ± 11.1), regardless of the technique of skin closure. Almost no correlation at all was observed between age and total PSAS score (*r* = 0.056).

Cronbach alpha for PSAS was 0.84 and the correlation between total score and the score of the overall opinion was 0.78.

A mild but significant correlation between OSAS and PSAS total score was also observed (Pearson *r* = 0.54).

## 4. Discussion

When considering a cervical incision, the aesthetic outcome is considered highly significant, since the wound is almost permanently on view. This aspect becomes furthermore important if we consider that young women constitute a large proportion of patients affected by a thyroid disease. In recent years, surgeons have become increasingly interested in obtaining an optimal aesthetic outcome. 

Minimally invasive thyroidectomy techniques have been developed in an effort to improve aesthetic results as well as minimizing pain and shortening hospital stay. A shorter incision, however, does not necessarily confer an improvement of patient overall satisfaction and opinion on aesthetic outcome [22, 23]. In this context, the choice of the method of suture can be a critical factor in the scar appearance.

We found that subcuticular suture gives a better aesthetic outcome than octyl-cyanoacrylate when the scar is assessed by a medical observer in an early postoperative phase. This difference disappeared when the self-assessment of patients was considered. 

Many of previous studies comparing tissue adhesive with other techniques in neck surgery relied on a simple mono-dimensional numeric scale of measure of patient's satisfaction [5, 10, 24, 25]. Only Pronio et al. [12], Lombardi et al. [14], and Ong et al. [26] used validated instruments to express the surgeon's assessment of the scar, but only the first two studies investigated neck wounds and were in some way comparable to our study. Pronio et al. [12] did not observe any difference between metal clips and tissue adhesive, while Lombardi et al. [14] reported a worse score for tissue adhesive, with respect to subcuticular suture or metal clips, both using a modified Vancouver scale and PSAS. These two studies used different instruments to evaluate the quality of scars and this could be a possible explanation for their contradictory results. Another possible explanation could be the different frequency of the minor complications related to the use of cyanoacrylate, more in particular bleeding from dermal margins at the moment of application of glue. This kind of bleeding may occur at the end of the operation, when the hyperextended position of the neck is relaxed and the anesthetic gas supply is closed, so blood pressure tends to rise up to its normal level and dermal blood flow is restored to its normal distribution. We had 4 (16%) cases of mild bleeding from the dermal margin, more frequently than Pronio reported, and this could have prevented a perfect alignment of margins, leading to a poor evaluation six weeks later. Multivariate analysis indicated that hyperpigmentation and relief were the factors that mostly influenced observer's judgment, and this finding could be coherent with an imperfect alignment of margins, possibly resulting in a flow of glue in the wound or a scaled scar.

The observed difference between the OSAS and the PSAS score is in agreement with some recurrent findings of the literature [22, 23, 27] that tend to undervalue the influence that the appearance of the scar has on patient's satisfaction, when compared to technical medical judgment or to the assessment of naive viewers [22].

An important consideration deserves the high variability in PSAS score, witnessed by the wide range of the standard deviation. Despite that the internal consistency of the instrument was good (Cronbach alpha = 0.84), it is clear that criteria by which patients assess their scar are highly subjective, various, and difficult to capture [28], resulting in a weak intersubjective comparability of judgment. Pain, color, and thickness were the significant contributing factors to the overall satisfaction at multivariate analysis. It is noteworthy that while the latter two are directly related to the esthetic outcome, pain is a general symptom and for a patient may be difficult to discriminate if pain comes from the scar of the underlying site of the operation (muscles and fascial layer).

Many factors contribute to the determination of patient's satisfaction with surgery related to cultural context and outcome [29] as well as personal factors, like the expectation and the familiarity with what a surgeon would rate as a “normal” outcome [30]. Setting a good communication with the patients and their families and allowing to share a feasible expected outcome [31] could probably be as important as achieving a smaller access and better scars.

Tissue adhesive was advocated to decrease pain and discomfort, since many patients are anxious at the prospect of removal of sutures and occasionally describe it as of more concern than the procedure itself [32]. This aspect can influence patient's overall satisfaction. In our study subcuticular suture was performed with absorbable material, so removal of stitches was not required, as for glue. 

This study has some limitations. First, it is a monoinstitutional study; hence, our results could be influenced by local expertise and habits, even if we were already using both octyl-cyanoacrylate and subcuticular suture for many years, so there was no possible learning curve effect. A second possible limitation derives from the interruption of the trial at the check point of interim analysis. Although the difference in OSAS was significant and for that difference the computed power of the assessed sample was 85%, limiting the number of patients could have prevented to uncover a difference in PSAS too. A third limitation is that our followup is limited to 6 weeks, but it is known that the appearance and the assessment of scars tend to get better as time goes on [14]. A final limitation is that each patient was assessed by only one observer, but the two observers had a period of training to reach a high consistency in their judgment. 

We did not measure the time needed for closure but the duration of the overall surgical procedure was not different between the two groups and, in any case, the duration of the phase of approximation of dermal margins in itself is irrelevant with respect to the whole duration of the operation, especially when considering the short length of the wound (Table 1). Furthermore, though not within the remit of this study, it is likely that subcuticular suture can be achieved more economically than glued closure [14, 33].

## 5. Conclusion

Subcuticular suture is often considered to be a technique which requires a particular surgeon's expertise and a longer time of execution, even if it has been indicated as the gold standard for neck surgery [14] and a recent systematic review could not find difference in the duration of the procedure [8].

This study demonstrated that the use of subcuticular absorbable suture in the short term had a better cosmetic outcome than the use of tissue glue. This observation in association with the favorable rating of patients led us to consider absorbable subcuticular suture as the standard method of closure. We planned a long-term follow-up study at one year to confirm this finding.

However, it is important to consider that a correct technique is fundamental to have a good cosmetic appearance of the scar, whatever the chosen method is, and that patients' satisfaction relies on many more factors than just the aesthetic appearance of the scar.

## Figures and Tables

**Table 1:** Personal and clinical data of patients.

	Octyl-cyanoacrylate (25 pts)	Subcuticular suture (25 pts)
Sex		
F	15 (60%)	18 (72%)
M	10 (40%)	7 (28%)
Age (yrs)^1^	52.7 (14.2)	59.2 (12.6)
Diagnosis		
Benign	19 (76)	18 (72)
Tumor	6 (24)	7 (28)
Length of wound (cm)^1^	4.2 (0.8)	4.0 (0.7)
Duration of operation (min)^1^	87.0 (13.2)	94.5 (13.5)
Bleeding from wound margins	4 (16)	0 (0)
Wound complications (infection and dehiscence)	0 (0)	0 (0)

Sex, diagnosis, bleeding, and complications are expressed as number (%).

Age, length of wound, and duration of operation are expressed as mean (±st. dev.).

^1^ *t*-test not significant.

**Table 2:** Distribution of mean score (±st. dev.) for the items of OSAS, stratified for type of closure.

Type of closure		Vascular score	Pigmentation score	Thickness score	Relief score	Pliability score	Surface score	Total score	Overall opinion score
Octyl-cyanoacrylate	Mean	2.53	2.65	2.06	2.24	2,18	1.65	13.29	2.71
	St. dev.	1.0	1.2	0.8	0.80	1,0	0.6	4.4	1.1

Subcuticular suture	Mean	1.72	1.72	1.72	1.61	1,78	1.61	10.17	2.06
	St. dev.	0.7	0.8	0.7	0.7	1,0	0.8	3.8	0.9

Mann-Whitney *U* test for independent samples.

Vascular, pigmentation, and relief score *p* < 0.03; total score *p* = 0.038; all the other scores: not significant. OSAS: Observer Scar Assessment Scale.

**Table 3:** Multivariate regression models of the relationship among OSAS and PSAS items and the overall opinion score.

	*β* standardized coefficient	*p*
OSAS-corrected *R* ^2^ = 0.71		
Pigmentation score	0.52	0.000
Relief score	0.43	0.001
PSAS-corrected *R* ^2^ = 0.61		
Pain	0.26	0.025
Color	0.43	0.007
Thickness	0.35	0.045

OSAS: Observer Scale; PSAS: Patient Scar Assessment Scale.

**Table 4:** Distribution of mean score (±st. dev.) for the items of PSAS, stratified for type of closure.

Type of closure		Pain score	Itching score	Color score	Stiffness score	Thickness score	Irregularity score	Total score	Overall opinion score
Octyl-cyanoacrylate	Mean	0.59	1.65	2.71	1.76	2.06	2.24	11.00	1.65
	St. dev.	1.1	2.5	2.7	2.4	2.6	2.4	9.8	1.6

Subcuticular suture	Mean	1.44	1.50	2.56	1.56	1.67	2.72	11.44	2.17
	St. dev.	2.3	2.2	2.2	2.5	2.2	2.6	11.5	2.0

Mann-Whitney *U* test for independent samples: no significant differences in mean.

PSAS: Patient Scar Assessment Scale.
